# Supplementary material for: Clostridioides difficile major toxins remodel the intestinal epithelia, affecting spore adherence/internalization into intestinal tissue and their association with gut vitronectin
Source: bioRxiv. 2025 Jan 29:2025.01.29.635439. Preprint. [Version 1] doi: 10.1101/2025.01.29.635439 (PMC11838273; doi:10.1101/2025.01.29.635439)
Supplement: 1 [file NIHPP2025.01.29.635439V1-supplement-1.pdf]

bioRxiv preprint doi: https://doi.org/10.1101/2025.01.29.635439; this version posted January 29, 2025. The copyright holder for this preprint (which was not certified by peer review) is the author/funder, who has granted bioRxiv a license to display the preprint in perpetuity. It is made available under aCC-BY 4.0 International license.

Fig. S1 | Effect of TcdA/TcdB intoxication of intestinal epithelial Caco-2 cells in levels of Fibronectin and Vitronectin. Differentiated Caco-2 cells intoxicated with TcdA and TcdB for 3, 6, or 8h in DMEM FBS-free. As a control, cells were treated with DMEM FBS-free. Relative fluorescence intensity measured as the sum of raw intensity density/area for each z-step of accessible **a** (acc) and total Fn, its abundance in the cell; in the same way, the relative fluorescence intensity of **b** (acc) and total Vn, its abundance in the cell. Controls were set at 100%. Error bars indicate the mean ± SEM from at least 9 fields ($n = 3$). Statistical analysis was performed by Two-Way ANOVA post-Bonferroni; ns, $p > 0.05$; * $p < 0.05$.

bioRxiv preprint doi: https://doi.org/10.1101/2025.01.29.635439; this version posted January 29, 2025. The copyright holder for this preprint (which was not certified by peer review) is the author/funder, who has granted bioRxiv a license to display the preprint in perpetuity. It is made available under aCC-BY 4.0 International license.

Fig. S2 | TcdA and TcdB increase accessible α_5 and α_V but no β_1 integrins in intestinal epithelial cells. Differentiated Caco-2 cells intoxicated with 600pM of TcdA and TcdB for 8h in DMEM FBS-free. As a control, cells were treated with DMEM FBS-free. Unpermeabilized cells were stained for accessible integrin (green), permeabilized, and stained total integrin (red) and F-actin (grey). Cells were immunostained for α_v integrin; Relative fluorescence intensity measured as the sum of raw intensity density/area for each z-step of **a,** acc α_5 and total α_5 in cells, **b**, acc α_V and total α_V in cells,**c,** acc β_1 and total β_1 in cells. **d, e, f**, Representative confocal microscopy images 3D projection of control cells (left) and intoxicated cells for 8h (right); below 4x magnified slides (XY), and the orthogonal view (XZ). Controls were set 100%. Error bars indicate the mean ± S.E.M from at least 9 fields (n = 3). Statistical analysis was performed by unpaired Student's t test, ns indicates non-significant differences; ** $p < 0.01$; **** $p <$ <0.0001. Bars, top panels 20 µm; bottom panels 5µm.

bioRxiv preprint doi: https://doi.org/10.1101/2025.01.29.635439; this version posted January 29, 2025. The copyright holder for this preprint (which was not certified by peer review) is the author/funder, who has granted bioRxiv a license to display the preprint in perpetuity. It is made available under aCC-BY 4.0 International license.

Fig. S3 | Effect of TcdB-intoxicated ileal loop in accessible and total levels of Fibronectin and Vitronectin. Ileal ligated loops were intoxicated for 5 h with 0.1, 0.5, 1, or 5µg of TcdB or saline as control. Then loops were removed, washed, fixed, and subjected to immunofluorescence. Unpermeabilized tissues were stained for accessible Fibronectin or Vitronectin (acc Fn or acc Vn; green), and then permeabilized and stained total Fibronectin or Vitronectin (total Fn or total Vn; red) and F-actin (grey). **a-b**, Representative confocal microscopy images 3D projection of intoxicated loops for 5 h with 0.1µg (left), intoxicated loops for 5 h with 0.5µg (middle), and intoxicated loops for 5 h with 0.1µg (right) immunostained for accessible and total Fn or Vn; right bottom, a magnified 3D projection, next to a magnified z-stack (XY), and then magnified orthogonal view (XZ). Scale bar 20 µm.

Figure S1

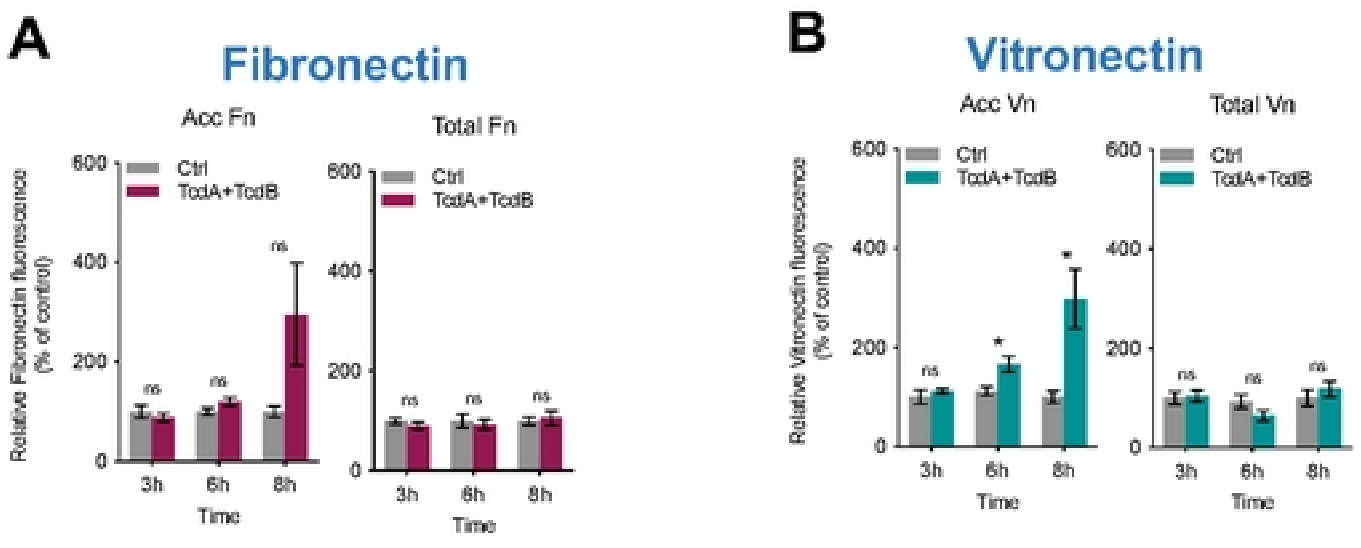

bioRxiv preprint doi: https://doi.org/10.1101/2025.01.29.635439; this version posted January 29, 2025. The copyright holder for this preprint (which was not certified by peer review) is the author/funder, who has granted bioRxiv a license to display the preprint in perpetuity. It is made available under aCC-BY 4.0 International license.

Figure S1

Figure S3

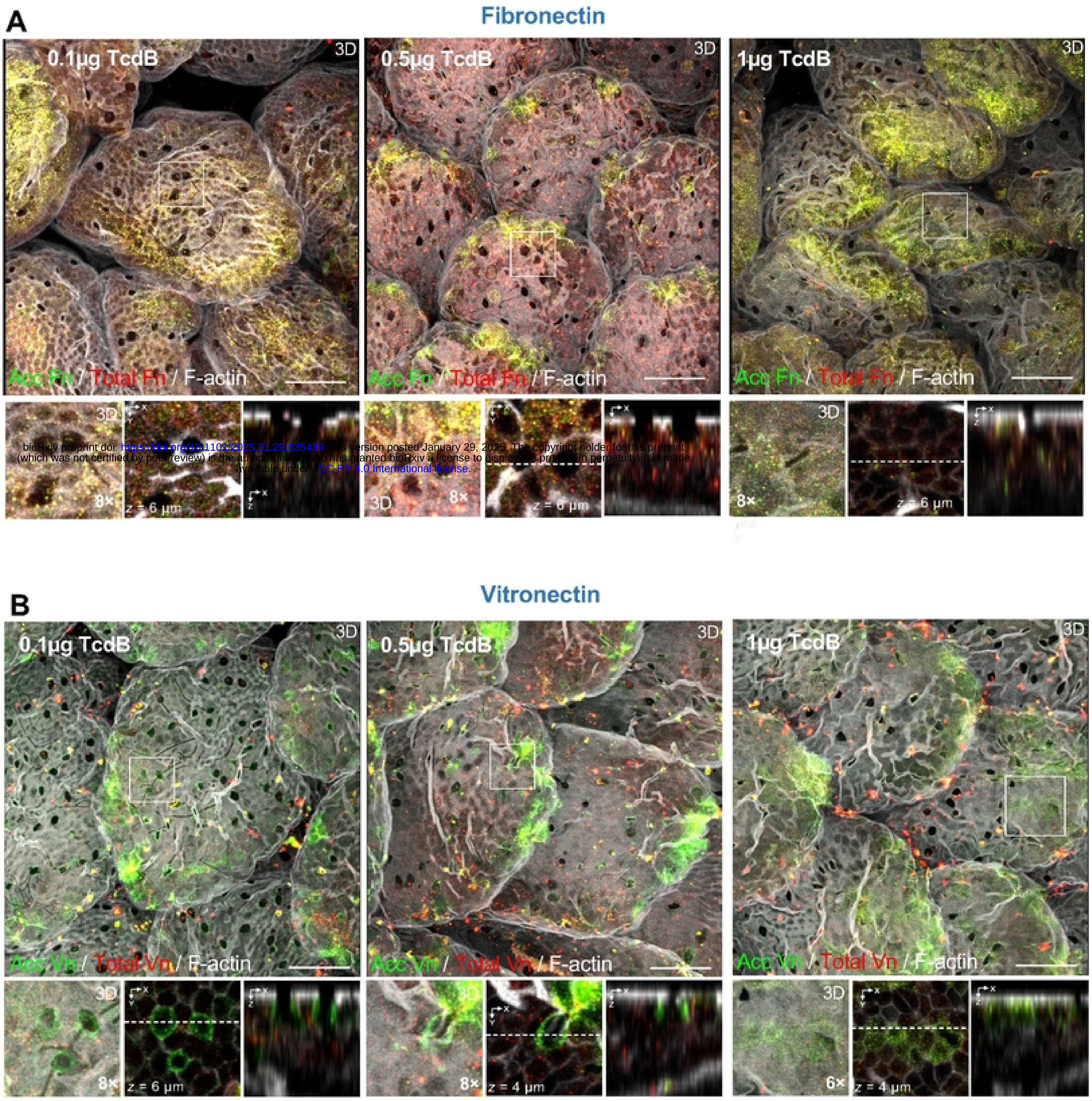

bioRxiv preprint doi: https://doi.org/10.1101/2025.01.29.635439; this version posted January 29, 2025. The copyright holder for this preprint (which was not certified by peer review) is the author/funder, who has granted bioRxiv a license to display the preprint in perpetuity. It is made available under a CC-BY 4.0 International license.

Figure S3

Figure S2

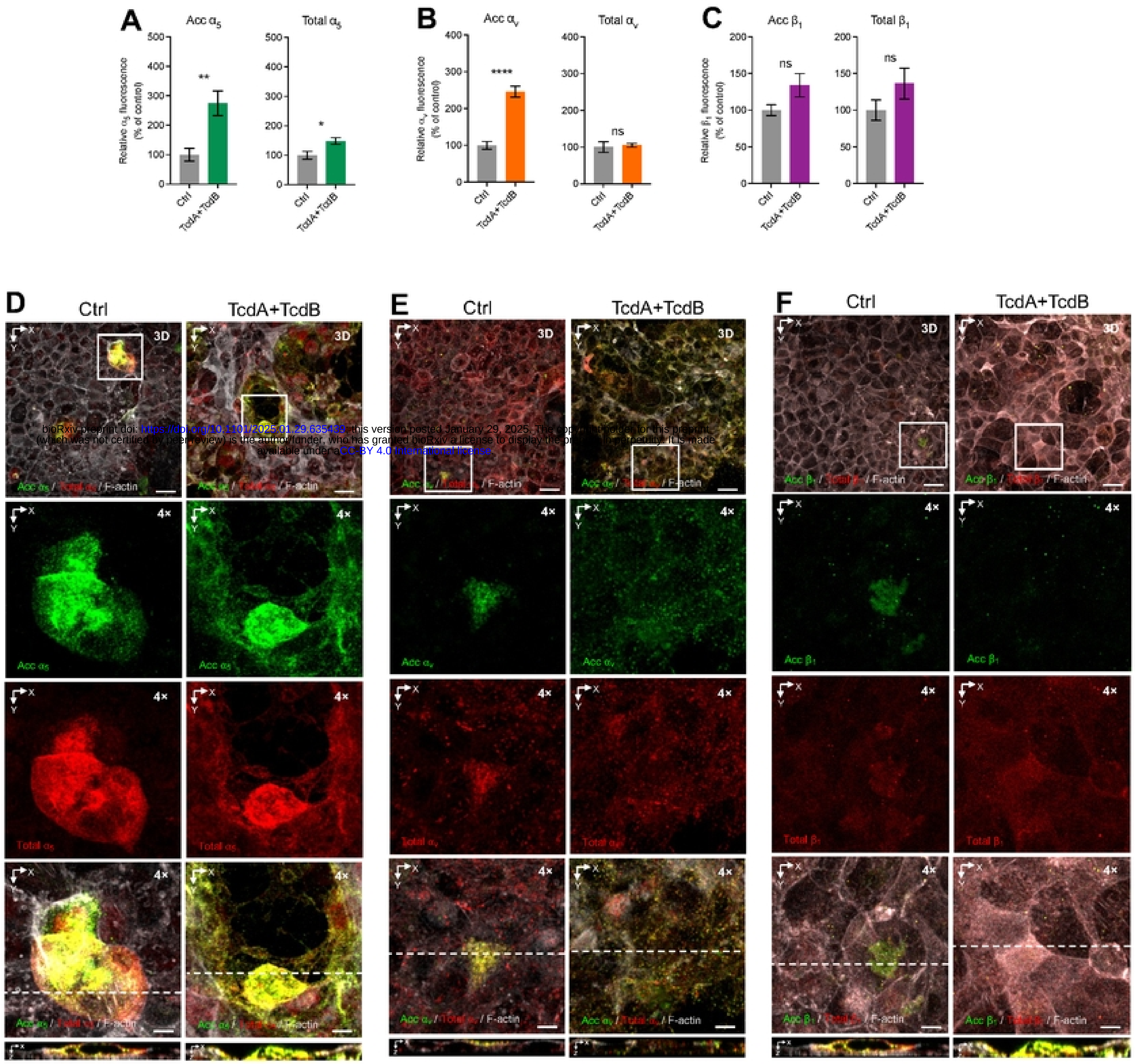

bioRxiv preprint doi: https://doi.org/10.1101/2025.01.29.635435; this version posted January 29, 2025. The copyright holder for this preprint (which was not certified by peer review) is the author/funder, who has granted bioRxiv a license to display the preprint in perpetuity. It is made available under aCC-BY 4.0 International license.

Figure S2
